# Corrosion Behavior and Ion Release of Co–Cr Dental Alloys Fabricated by Casting, CAD/CAM, SLM and DMLS: Influence of Manufacturing Route and Microstructure

**DOI:** 10.3390/bioengineering13040406

**Published:** 2026-03-31

**Authors:** Lucien Reclaru, Gabriel Buciu, Stelian-Mihai-Sever Petrescu, Raluca Ionela Gheorghe, Daniela Florentina Grecu, Alexandru Florian Grecu

**Affiliations:** 1 Scientific Independent Consultant Biomaterials and Medical Devices, 2074 Marin-Neuchâtel, Switzerland; 2 Faculty of General Health Care, Titu Maiorescu University, Bd. Ecaterina, Teodoroiu No. 100, 210106 Targu Jiu, Romania; 3 Department of Orthodontics, University of Medicine and Pharmacy of Craiova, Street Petru Rareș 2, 200349 Craiova, Romania; 4 Dental Medicine, University of Medicine and Pharmacy of Craiova, Street Petru Rareș 2, 200349 Craiova, Romania; 5 Filantropia Municipal Clinical Hospital of Craiova, 200349 Craiova, Romania; 6 Department of Orthopedics and Traumatology, University of Medicine and Pharmacy of Craiova, 200349 Craiova, Romania; alexandru.grecu@umfcv.ro

**Keywords:** Co–Cr alloys, powder Co–Cr, casting, CAD/CAM, powder CoCr, 3D printing SLM, DMLS, microstructure, rotating electrode, electrochemical evaluation, polarization curves, crevice corrosion, cation release, Mann–Whitney (MW) test

## Abstract

The present study demonstrates that the corrosion behavior of dental cobalt–chromium (Co–Cr) alloys is strongly influenced by the interaction between microstructure, manufacturing technique, and oral chemical environment. A comparative investigation was conducted on Co–Cr specimens fabricated using four technological routes: conventional casting, CAD/CAM machining, Selective Laser Melting (SLM), and Direct Metal Laser Sintering (DMLS). The study included microstructural characterization, evaluation of generalized corrosion behavior using the rotating electrode technique, assessment of localized crevice corrosion, and quantitative analysis of the release of twenty metallic cations. Extraction tests were performed for 168 h in two media simulating aggressive oral environments: 0.07 N HCl (acidic medium) and a fluoride-containing electrolyte (0.1% NaF + 0.1% KF). Electrochemical measurements were recorded in the current density range of 10^−10^ to 10^−7^ A/cm^2^, while released cation concentrations were quantified at the µg/L level. All alloys exhibited very low corrosion current densities (icorr in the 10^−8^ to 10^−9^ A·cm^−2^ range), confirming overall good corrosion resistance. Among all manufacturing routes, CAD/CAM specimens demonstrated the highest electrochemical performance, with a wide passivity domain extending up to approximately 740 mV/SCE. A statistical interaction analysis between extraction media and manufacturing techniques was performed using the non-parametric Mann–Whitney (MW) U test. Among the analyzed elements, only chromium showed a statistically significant difference between media (*p* < 0.05), with an approximately 25-fold-higher release in acidic conditions compared with the fluoride medium, confirming the predominant role of proton-induced destabilization of the protective Cr_2_O_3_ passive film. In contrast, fluoride-containing media induced selective release of elements such as Cu (3× higher), W (2.5× higher), and Mo (1.4× higher), associated with complexation phenomena. The manufacturing route significantly influences corrosion behavior. Although additive manufacturing technologies (SLM/DMLS) enable highly accurate and customized prosthetic designs, rapid solidification and microstructural heterogeneities may increase susceptibility to localized corrosion compared with more homogeneous CAD/CAM materials. Clinically, these findings suggest that future restorative strategies should incorporate corrosion-aware material selection within digital workflows. As digital dentistry evolves, predictive models integrating patient-specific oral conditions may assist clinicians in selecting the most appropriate material system for long-term performance. In conclusion, the long-term success of dental Co–Cr prosthetic devices depends not only on mechanical strength and precision of fit, but also on sustained electrochemical stability in the complex oral environment.

## 1. Introduction

Over the past three decades, dentistry has undergone a major transformation driven by the integration of digital technologies in diagnosis, treatment planning, and prosthetic manufacturing. Among these advances, computer-aided design and manufacturing (CAD/CAM) and, more recently, additive manufacturing (3D printing) have significantly reshaped the design, production, and performance of dental biomaterials [1,2,3]. In parallel, bioprinting has emerged as a promising approach toward biologically functional structures [4,5]. CAD/CAM technologies have enabled the fabrication of dental restorations with high dimensional accuracy and reproducibility [1,2]. Materials such as cobalt–chromium (Co–Cr) alloys, titanium alloys, and ceramics exhibit controlled microstructures and reliable mechanical properties when processed by subtractive methods [3]. However, these approaches remain limited by material waste and geometric constraints, which has driven the rapid development of additive manufacturing techniques such as Selective Laser Melting (SLM) and Direct Metal Laser Sintering (DMLS) [6,7,8,9].

Additive manufacturing introduces specific microstructural features, including fine cellular or dendritic morphologies, high solidification rates, and metastable phase distributions [9,10]. These characteristics strongly influence mechanical behavior, corrosion resistance, and ion release kinetics. Comparative studies between cast and additively manufactured Co–Cr alloys have reported significant differences in density, microstructure, and electrochemical performance [9,11], while the clinical adaptation of SLM/DMLS frameworks has shown promising but process-dependent outcomes [12,13].

Fluoride-containing environments may destabilize this passive layer and promote ion release [14]. In addition to uniform corrosion, localized phenomena such as pitting and crevice corrosion can further accelerate degradation and cation release, potentially affecting long-term biocompatibility [9,10].

Recent advances in digital workflows combining CAD/CAM, additive manufacturing, and guided implantology have improved precision and clinical outcomes in prosthetic dentistry [15,16]. Furthermore, emerging technologies such as 4D printing and bioprinting are expanding the field toward adaptive and biologically interactive materials [4,7,17,18,19,20,21]. Future developments are expected to focus on microstructural optimization, alloy design, and improved corrosion resistance, supported by advances in process control and material engineering [9,22].

On the horizon, this complementarity should intensify with the increasing integration of artificial intelligence. Design systems will become more intelligent, able to analyze the clinical, functional and biological data of the patient in order to propose optimized and personalized designs. In this context, 3D printing will establish itself as a natural extension of advanced CAD, allowing to materialize complex structures, multi-material and bioactive, difficult to achieve by conventional machining. 3D bioprinting pushes this evolution of the CAD/CAM and 3D printing paradigm even further. Bioprinting introduces the notion of biological computer-aided design. Future software will no longer be limited to geometric modeling, but will integrate biological parameters such as cell distribution, biomaterial composition, porosity, vascularization and mechanical gradients. Thus, CAD will become a living-tissue design tool, while bioprinting will represent an advanced form of biological CAM [23,24]. Despite the growing body of literature on individual manufacturing routes, a systematic head-to-head comparison of the corrosion behavior and multi-elemental cation release kinetics across all four major manufacturing pathways—conventional casting, CAD/CAM machining, SLM, and DMLS—using identical electrochemical protocols and clinically relevant extraction media remains absent from the literature. This gap limits the ability of clinicians to make evidence-based, corrosion-aware material selections within digital workflows. These advances will contribute to the emergence of a digital, personalized [25,26], and biologically integrated dentistry combining technological precision, physicochemical stability, and biological safety [27,28,29,30,31,32,33,34].

### 3D Printing Technologies in Dentistry

The development of 3D printing in dentistry is driven by the diversification of additive manufacturing technologies, each adapted to specific clinical, mechanical, and biological requirements. Modern digital dentistry relies on complementary processes selected according to material type, clinical indication, and required precision. These technologies enable the fabrication of customized devices with high geometric accuracy and controlled microstructure. Additive manufacturing processes can be broadly classified into metallic systems and polymer/resin-based systems.

For metallic biomaterials such as cobalt–chromium, titanium, and stainless steels, additive manufacturing relies on layer-by-layer fusion of metal powders using a concentrated energy source (laser or electron beam). Selective Laser Sintering (SLS), one of the earliest techniques, allows partial sintering of powders but may result in residual porosity and reduced density [34,35,36,37]. In contrast, Direct Metal Laser Sintering (DMLS) enables near-complete fusion, producing high-density components with mechanical properties comparable to bulk materials [38,39]. Selective Laser Melting (SLM) achieves full melting of powders under controlled atmosphere, leading to dense structures with refined microstructures due to high solidification rates [40,41,42]. Electron Beam Melting (EBM), performed under vacuum, also produces fully dense parts while reducing residual stresses, making it suitable for implant applications and porous structures promoting osseointegration [43,44]. 

In dentistry, these technologies allow the manufacture of numerous metal structures, notably: removable prosthesis frames, crowns and bridges, plantar bars, personalized implant pillars, complex implant infrastructures, personalized maxillofacial devices [45,46,47].

The high precision of these technologies allows their full integration into digital CAD/CAM workflows. The future developments are anticipated to include: microstructur-al optimization of metal alloys, improvement of biocompatibility, fabrication of mul-ti-material structures and integration of bioactive materials, and the development of bio-mimetic structures favoring biological integration [48,49,50,51,52,53,54,55,56,57].

The objective of the study was an in-depth comparative analysis focusing on: the characterization of the microstructures obtained according to each development process, the evaluation of the resistance to generalized corrosion, the morphological analysis of the localized crevice corrosion, and the study of the release kinetics of twenty metallic cations. The salting-out tests were carried out in two extraction media simulating aggressive environments relevant to dentistry: a 0.07 N HCl solution (acidic medium) and a fluorinated electrolyte composed of 0.1% NaF + 0.1% KF.

A comparative statistical analysis was conducted to evaluate the interaction between the two extraction environments and the different development pathways. A non-parametric Mann–Whitney U test was applied to determine the significance of differences observed between groups. The results obtained make it possible to establish correlations between the manufacturing process, microstructure, electrochemical behavior and kinetics of ion release. They also offer reflections on the prospects for the evolution of additive and subtractive manufacturing technologies in the field of dental biomaterials, with a view to improve their electrochemical stability and long-term biological safety.

## 2. Materials and Methods

### 2.1. Chemical Composition

For our study, we used e grades of CoCr alloys that show different compositions corresponding to their respective manufacturing techniques. This reflects the real clinical scenario, in which each manufacturing route is commercially associated with specific, optimized alloy formulations; practitioners do not use a single identical alloy composition across all four processes. Thus, our experimental design was conceived to compare the corrosion performance of materials as they are actually used in clinical practice, maximizing translational relevance. The compositional differences—particularly the presence of tungsten in certain alloys and variations in Mo and Cr content—are acknowledged as a confounding variable and are explicitly considered in the interpretation of cation release data. Table 1 shows the composition of the CoCr alloys used to prepare the samples for the corrosion behavior and cation release evaluation tests.

The presence of trace copper in stainless steels, NiCr, CoCrMo, and refractory alloys is a problem at the global steel industry level. It is a permanent pollution from the production process that originates from metal waste coming from certain geographical areas. Today, purification by oxidation in cast iron crucibles is no longer sufficient, and secondary vacuum casting remains costly. 

#### 2.1.1. General Scientific Comments on CoCr Alloy Diagram (Figure 1) [58,59,60,61]

**Figure 1:**
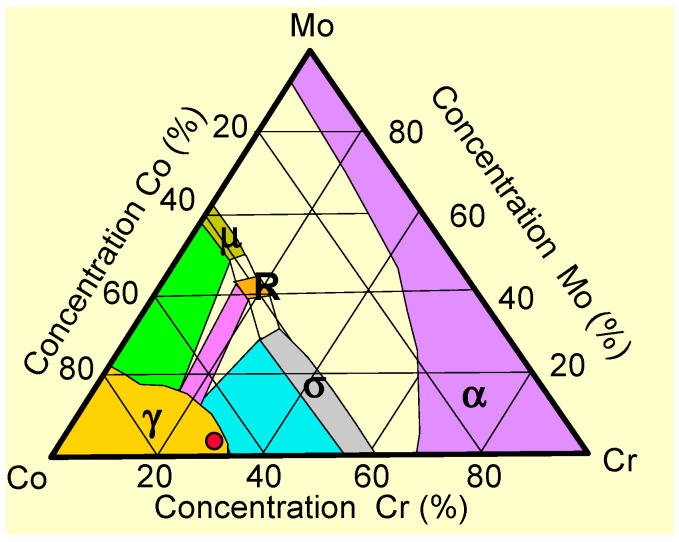
Ternary Co–Cr–Mo phase diagram illustrating the distribution of γ, ε, σ, and α phases as a function of alloy composition.

In Figure 1, the ternary diagram of CoCrMo alloys is shown. These alloys reveal a very complex multiphase structure. Below are some scientific comments regarding this type of structure. The ternary Co–Cr–Mo phase diagram highlights the strong influence of chemical composition on phase stability and microstructural domains relevant to dental alloys. The compositional regions represented illustrate the coexistence of multiple crystallographic phases, including γ (FCC), ε (HCP), σ, and α phases, which are known to significantly affect the mechanical behavior, corrosion resistance, and ion release kinetics of Co–Cr-based dental materials.

‑ The γ-phase domain, located in the Co-rich region, is typically associated with enhanced ductility and toughness, making it particularly favorable for dental prosthetic frameworks subjected to cyclic loading. In contrast, the ε phase appears at intermediate Cr and Mo contents and is often linked to increased hardness but reduced ductility, potentially influencing fatigue behavior.
‑ The presence of the σ phase, especially in Cr- and Mo-enriched regions, is of particular concern from a clinical perspective. This brittle intermetallic phase is known to degrade both mechanical performance and corrosion resistance, promoting localized corrosion phenomena and potentially accelerating cation release under oral conditions.
‑ The α phase, observed at high chromium concentrations, reflects the stabilizing effect of Cr on corrosion resistance through passive oxide layer formation. However, excessive Cr enrichment may compromise microstructural homogeneity, especially in additively manufactured components.
‑ When tungsten (W) is added to the alloy composition, as a refractory element, several structural modifications are expected to occur. These modifications contribute to (a) stabilization of the γ phase (FCC) of cobalt, (b) expansion of the γ-FCC-phase domain at the expense of the ε-HCP phase and partially the σ phase, (c) the formation of a more stable and durable microstructure, and (d) enhanced γ-phase stability at high temperatures, which explain its frequent incorporation into powder compositions used for additive manufacturing (3D printing). Tungsten thus plays a critical role in improving the high-temperature stability, phase balance, and overall structural integrity of Co–Cr alloys processed by advanced manufacturing techniques. For a clearer understanding of these mechanisms, a comprehensive synthesis of the discussed concepts is presented in Table 2.

From a future-oriented perspective, this diagram underscores the need for precise compositional control in next-generation digital dentistry workflows. Optimizing Co–Cr–Mo compositions to remain within single-phase or corrosion-resistant multiphase domains will be critical for improving long-term biocompatibility and reducing ion release in additively manufactured dental implants and prostheses: γ (gamma) phase–FCC (Face-Centered Cubic) and ε (epsilon) phase–HCP (Hexagonal Close-Packed).

Interpretation of phase zones:

**Table 2:** Summary of phase characteristics from the Co–Cr–Mo diagram (Figure 1).

Phase	Phase Structure	Mechanical Effect	Corrosion Behavior	Clinical Relevance
γ	FCC (Face-Centered Cubic), stable; cobalt-rich (Co)	Ductile, tough	Very good	High
ε	HCP (Hexagonal Close-Packed), metastable; martensitic transformation γ → ε	Harder, less ductile	Moderate	Moderate
σ	Intermetallic phase, complex tetragonal structure, stable; rich in Cr and Mo	Brittle	Poor	Unfavorable
R	Rhombohedral phase, metastable; enriched in Mo, with Cr and Co	Less brittle than σ phase	Vers unfavorable	Very unfavorable
µ	Intermetallic phase, complex hexagonal structure, metastable; very rich in Mo	Brittle	Poor	Very unfavorable

#### 2.1.2. Co–Cr Powder for 3D Printing

**Figure 2:**
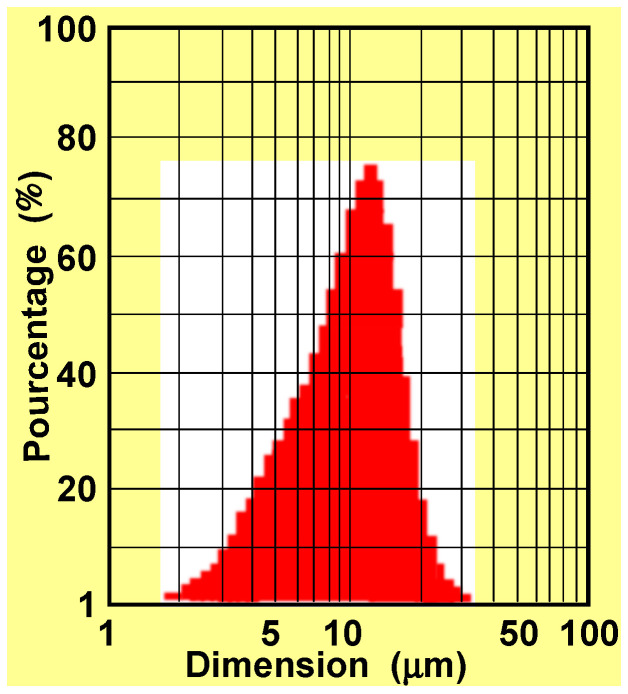
Particle size distribution of the Co–Cr powder used for additive manufacturing.

The particle size distribution of the Co–Cr powder exhibits a unimodal and relatively narrow distribution, characteristic of gas-atomized powders intended for Laser Powder Bed Fusion (LPBF). The majority of particles are concentrated within the 5–20 µm range, with a pronounced peak around 10–12 µm, indicating good process control during powder atomization. The absence of significant secondary peaks suggests a limited presence of fine (<5 µm) and coarse particles (>30 µm), which is favorable for achieving consistent powder flowability and homogeneous layer deposition during the 3D printing process. (Figure 2). From a material performance perspective, the observed particle size distribution supports the formation of a fine cellular microstructure with rapid solidification rates. While this microstructure is advantageous for mechanical strength, it may also promote elemental segregation at cell boundaries, particularly for Mo and Cr, which has been associated with increased susceptibility to localized corrosion and cation release if not properly mitigated by post-processing treatments.

For dental applications, this powder size distribution is well aligned with the requirements of LPBF-manufactured prosthetic frameworks, enabling high geometric accuracy, low porosity levels, and good surface finish after post-processing. Nevertheless, long-term biocompatibility remains strongly dependent on powder lifecycle management, as repeated reuse of fine particles may intensify oxidation and affect corrosion behavior in the oral environment.

In the future, the optimization of Co–Cr powders for digital dentistry should focus on: chemically stabilized powders limiting the formation of μ/R/σ phases, custom-made alloys positioned mainly in single-phase γ areas of the Co–Cr–Mo diagram, advanced powder traceability (number of cycles, oxidation, segregation), and intelligent thermal treatments aimed at deactivating the γ/μ/R/σ pathways.

### 2.2. Sample Preparation

‑ Sample Preparation

Three types of specimens were prepared depending on the experimental analysis: disks (Ø 10 mm) for electrochemical corrosion tests, cylinders (Ø 5 mm × 20 mm) for crevice corrosion, and plates (14 × 35 mm) for ion release experiments. All samples underwent a standardized surface preparation procedure. Grinding was performed using SiC abrasive papers with progressively finer grit sizes (320, 500, 1200, and 1400) under running water to minimize thermal effects and remove debris. Final polishing was carried out using diamond suspensions (6, 3, and 1 μm; Struers, Copenhagen, Denmark) until a mirror-like surface finish was achieved. Samples were then ultrasonically cleaned in acetone (10 min), rinsed in deionized water (18 MΩ·cm, 10 min) followed by ethanol, air-dried, and stored in a desiccator prior to testing. For each manufacturing route, at least four independent specimens were prepared for ion release experiments (n = 4), while electrochemical and crevice corrosion tests were performed in duplicate. Results are reported as mean values.

‑ Manufacturing Routes and Materials

Four manufacturing approaches were investigated:

‑ Casting (Code #1): Co–Cr dental alloy fabricated by conventional casting using commercially available materials commonly employed in dental laboratories.
‑ CAD/CAM (Code #2): Samples machined from a Co–Cr disk (98 mm diameter, 12 mm thickness) supplied by a European manufacturer. Fabrication was performed using a 5-axis CNC milling system, enabling high precision and reproducibility.
‑ Additive manufacturing (Codes #3 and #4): Samples were produced by powder bed fusion using Co–Cr alloy powders compliant with ISO 22674:2022 (Type 5) [62]. The powder (particle size: 10–45 μm, density ≈ 8.7 g/cm^3^) was supplied by a specialized European manufacturer. It should be noted that, in industrial practice, alloy compositions are optimized for each manufacturing process. As a result, slight compositional differences may exist between casting, CAD/CAM, and additive manufacturing materials (Table 1). These differences are inherent to process requirements and may influence microstructure, phase stability, and corrosion behavior.

The powders used correspond to typical Co–Cr–Mo or Co–Cr–Mo–W systems (ASTM F75 [63], ISO 5832-4 [64]). In the present study, a tungsten-containing alloy (~5 wt%) was employed. Tungsten acts as a γ-phase stabilizer (FCC), promotes microstructural stability, and limits phase transformations (γ → ε), contributing to improved mechanical and corrosion properties.

All additive manufacturing samples were produced using virgin powder in order to avoid compositional drift and oxidation effects associated with powder recycling, which may induce heterogeneity and phase instability.

The powder used in the chemical composition contains 5% W. In the Co–Cr–W (or Co–Cr–Mo–W) phase diagram, tungsten plays a similar but more prominent role than Mo. Thus it is a refractory element (very high melting temperature). The stabilizer of the γ phase (FCC) of cobalt is strongly γ-gene at high temperatures, has slow diffusion with lasting microstructural effects, and widens the γ-FCC domain, at the expense of ε-HCP domains and partially σ. An element is said to be γ-gene when it stabilizes the FCC phase (γ), widens its stability domain, and again delays the γ– → ε transformation (HCP phase).

Two additive manufacturing processes were investigated: Selective Laser Melting (SLM, also referred to as Laser Powder Bed Fusion, LPBF) and Direct Metal Laser Sintering (DMLS).

### 2.3. Methodes

#### 2.3.1. Metallography

The samples were embedded in a cold-curing resin on a methyl methacrylate basis (Technovit, Kulzer, Friedrichsdorf, Germany), then polished with SiC paper (grit 320/500/1200/1400) and finally with diamond spray (6/3/1 micron) (Struers, Copenhagen, Denmark). Electrolytic etching was done in a bath of 100 mL H_2_O dest., 10 mL HCl conc. and 5 g chromium (VI)-oxide during 5 s under 0.4 V and 0.3 A according to the literature [9]. The microstructures of the alloys were observed using a metallographical microscope (Polyvar Met, Reichert-Jung, Vienna, Austria) and a scanning electron microscope (JEOL JSM 6300) equipped with an EDX system (Oxford, UK, INCA) for local phase analysis.

#### 2.3.2. Electrochemical Evaluation of Co–Cr Alloy Corrosion

Generalized corrosion was evaluated using a rotating disk electrode (RDE) in order to control mass transport conditions according to Levich theory [65,66]. This approach ensures a well-defined hydrodynamic regime and a controlled diffusion-layer thickness for electroactive species. To minimize the influence of dissolved oxygen, all experiments were conducted in a deaerated electrolyte using nitrogen purging, resulting in a residual oxygen concentration of approximately 0.2 mg·L^−1^. The electrode was operated under laminar flow conditions (Re ≈ 3200) at a rotation speed of 300 rpm.

**Figure 3:**
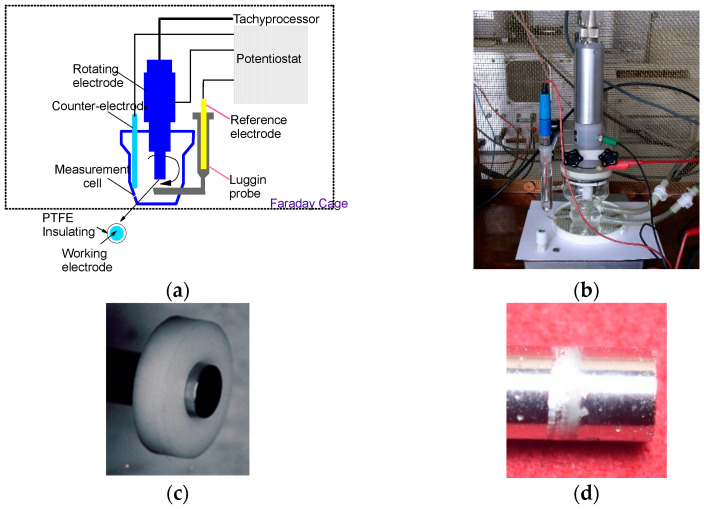
(**a**) Diagram of the rotating electrode technique; (**b**) rotating electrode used in the tests; (**c**) sample before test; (**d**) sample after test.

Electrochemical measurements were performed using a PAR Model 273A potentiostat in a glass corrosion cell adapted for RDE experiments. A platinum counter-electrode and a saturated calomel electrode (SCE) were used. The system was shielded within a Faraday cage, with a background noise of approximately 1 pA (Figure 3a,b).

The electrolyte consisted of Fusayama–Meyer artificial saliva (NaCl 0.4 g·L^−1^; KCl 0.4 g·L^−1^; NaH_2_PO_4_·H_2_O 0.69 g·L^−1^; CaCl_2_·H_2_O 0.79 g·L^−1^; urea 1.0 g·L^−1^). Tests were conducted at 37 °C and pH 5. The experimental protocol included the following:

‑ 16 h immersion at open circuit potential;
‑ Polarization scans (±150 mV) in the Tafel domain to determine (E_cor_) and (i_cor_);
‑ Tracing of the potentiodynamic polarization curves −1000 mV to +1000 mV vs. SCE;
‑ Analysis of localized corrosion and crevice corrosion.

Crevice corrosion tests were performed according to the ASTM F746-04 standard [67] in a 9 g·L^−1^ NaCl solution at 37 °C. Cylindrical samples (Ø 5 mm × 20 mm), polished to P1200, were mounted in polytetrafluoroethylene (PTFE) crevice assemblies (conical ring).

The test procedure consisted of cyclic potentiostatic steps. An initial anodic polarization at +800 mV vs. SCE was applied for 10 s, followed by a return to open circuit potential. The potential was then incrementally increased in steps of +50 mV, with each step held for 15 min. This sequence was repeated until a stable anodic current was observed. The crevice corrosion potential (Ecrev) was defined as the highest potential at which the current remained in the anodic domain.

The electrolyte was prepared using deionized water (18 MΩ·cm) and deaerated with nitrogen. The dissolved oxygen concentration decreased from 5.2–6.4 mg·L^−1^ to approximately 0.2 mg·L^−1^ after deaeration, as measured with the Viscolor Oxygen Kit S.A. 10 (Machery-Nagel-Düren, Germany). The pH remained stable at 5.7 before and after testing. Figure 3b,d illustrate the crevice assembly and the corrosion damage observed at the metal/PTFE interface, respectively.

#### 2.3.3. Release of Cations from CoCr Alloys

The release of cations was measured in the 0.07 N HCl (ISO DIN EN 71-3) [68] milieu and 0.1% NaF +0.1 KF % using Tracepur^®^ water and Ultrapur^®^ NaF end KF (Merck). The solutions were filtered before use over a sterilized Falcon 0.22 µm cellulose acetate membrane; the release flasks used were of Falcon sterile type made of polypropylene. The samples were of rectangular shape with dimensions 14 × 35 mm and were first cleaned in ethanol p.a. under ultrasound. The ratio of release solution volume/total sample surface was equal to 1. The extraction was carried out at 37 °C shielded from light for 168 h. Three samples of each CoCr alloy for each test environment were used. For each extraction environment, two blank samples were measured as a reference. The 0.07 N HCl medium was used in the evaluation of metals extracted from toys for children (ISO-DIN EN 71-3) [68]. Children have the tendency to put toys in their mouth, suck them and even swallow metallic pieces or plastic components. Children are more sensitive to toxicological reactions than adults. On the other hand, it is an excellent extraction medium without particular problems of preparation or quantitative analysis. The concentrations of 0.1% NaF and 0.1% KF are specific in the composition of toothpastes. Thus, it is interesting to evaluate the release of cations from CoCr alloys in this type of medium. The solutions were analyzed by two complimentary techniques, ICP-OES Optima 4300 Perkin Elmer and ICP MS Perkin Elemer. The cation matrices were measured according to the following scheme: ICP MS/Ba, Cd, Co, Hg, Hf, Mo, Nb, Pb, Sn, Sr, Zn; ICP OES/Cr, Cu, Fe, Ni, Ti, V, Zn; ICP OES Hydrides/As, Hg, Sb, Se. A statistical analysis of the released cations was performed. The maximum accepted risks for Type I error (false rejection of the null hypothesis, leading to the erroneous conclusion that differences between alloys exist) are: Fe (±22%), Cr, Cu, Mo, Ni, and Co (±17%) in 0.07 N HCl and Fe (±27%), Cr, Cu, Mo, Ni, and Co (±22%) in 0.1%NaF + 0.1%KF.

## 3. Results and Discussion

### 3.1. Microstructures

**Figure 4:**
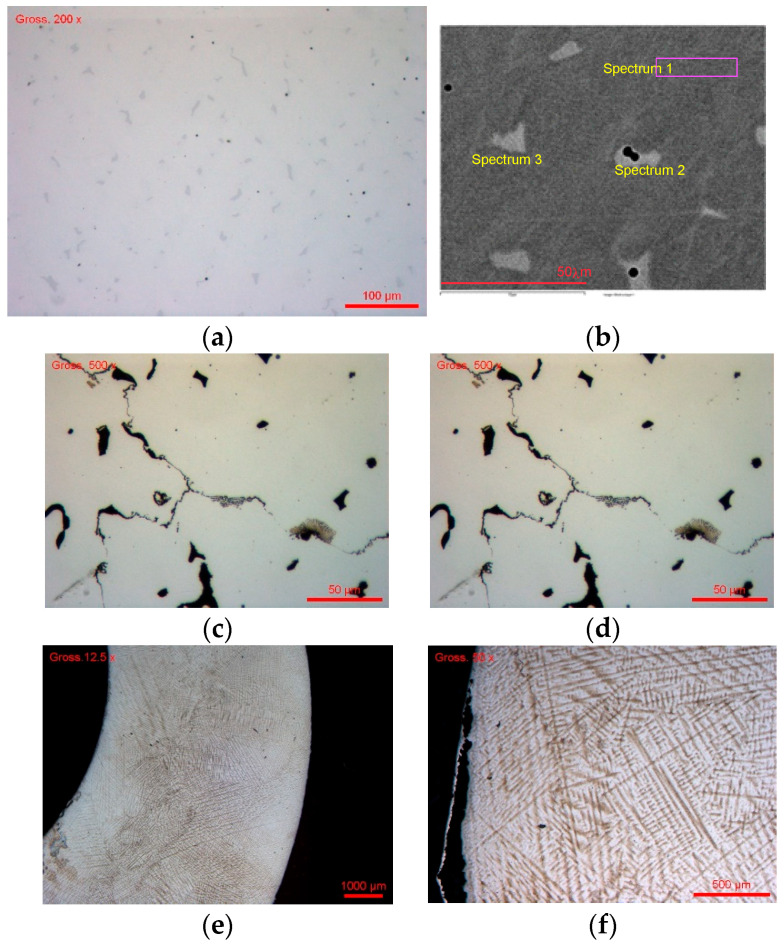
(**a**) Heterogeneous structure; (**b**) γ (FCC) matrix and secondary phases σ, µ and R with location of zones observed by EDX; (**c**) CoCr #1 after the chemical attack ×500; (**d**) CoCr #1 after the chemical attack × 1000; (**e**) microstructure before chemical attack; (**f**) microstructure after chemical attack.

For CoCr #1 obtained by the traditional casting technique (traditional casting process), the metallographic micrographs are shown in Figure 4a,b before the chemical attack and in Figure 4c,d after the chemical attack. Before attack, the micrographs (Figure 4a,b) show a contrasted two-phase microstructure, composed of: a continuous dark matrix, scattered white phases with an irregular morphology, and localized inclusions of distinct composition. This heterogeneity is typical of Co–Cr–Mo alloys resulting from solidification or thermomechanical fabrication, with elemental segregation. The EDX analysis of the chemical composition of the analyzed areas is presented in Table 3.

The spectra associated with the matrix (Spectrum 1) in Figure 4b and Table 3 show a dominant Co composition (~64 wt%), with Cr at ~28–29 wt%. This composition is characteristic of the cobalt-rich γ phase (FCC), which constitutes the ductile and majority phase of the Co–Cr alloy. The white phases (Spectrum 2) have a very different composition: high Cr (~38–39 wt%), high Mo (~23–24 wt%), and reduced Co (~35–37 wt%). This signature is typical of Cr- and Mo-rich intermetallic phases, attributable to σ (sigma) phases, μ (mu) phases, or R phases, depending on the thermal history. These phases are hard, fragile and electrochemically more noble, but can play a critical role in localized corrosion. The inclusions identified (Figure 4b and Table 3; Spectrum 3) show a high C and O content and the presence of Si, Cr, Co, Mo. This suggests complex oxides or contamination-fading residues, which can act as corrosion initiation sites.

After the chemical attack (the images of the structures are shown in Figure 4c,d), the alloy after chemical etching shows a clearly revealed microstructure compared to the non-etched state. We observe a preferential attack of the matrix, light relief areas, little or no attack, an accentuation of the matrix/secondary-phase interfaces, and the appearance of microcavities and grooves in the attacked areas. From a corrosion mechanistic standpoint, the dendritic segregation inherent to the casting process creates galvanic micro-cells between the Co-rich γ matrix and the Cr/Mo-enriched interdendritic zones. The compositional heterogeneity results in local differences in the electrochemical nobility of adjacent phases, promoting preferential dissolution of the anodic (Co-rich) matrix in the immediate vicinity of the cathodic (Cr/Mo-rich) secondary phases. This galvanic coupling mechanism is a primary driver of the localized corrosion observed in cast Co–Cr alloys and contributes to the higher cation release rates compared with more homogeneous microstructures.

*For sample #2*, the microstructures images show a dense and highly functional architecture, characteristic of prefabricated industrial disks intended for dental machining. Before chemical attack, the surface appears compact, free of macroscopic porosities, and without major microstructural discontinuities (Figure 4e). The chemical composition was determined by EDX at four different points on the sample surface after chemical attack (Figure 4f). The low variability of the measurements indicates good chemical homogeneity. The average composition (wt%) is 0.94 Si, 28.80 Cr, 0.88 Fe, 62.90 Co, and 6.48 Mo.

The following remarks may be made:

‑ The Cr content (~29 wt.%) is sufficient to ensure the formation of a stable passive Cr O film and excellent resistance to widespread corrosion.
‑ Mo (~6.5 wt.%) contributes to strengthening the resistance to localized corrosion, stabilizing the γ phase, and limiting the formation of σ/μ phases.
‑ The traces of Si and Fe, very weak, suggest a minor role, without any negative impact on biocompatibility.

After chemical attack (Figure 4f), the microstructural revelation highlights a continuous γ matrix (FCC) of dominant dendrites, with net but controlled grain boundaries and an absence of marked intergranular segregation, unlike cast alloys. This confirms that the CAD/CAM material comes from a strongly controlled industrial process (HIP, forging), favoring a thermodynamically stable microstructure.

### 3.2. Electrochemical Evaluation of Corrosion

‑ *Generalized corrosion*

The electrical potential of a metal immersed in an electrolyte varies over time but remains stable at a stationary value after a long period of immersion. This potential is not a characteristic of the metal. It depends on the experimental conditions, in particular the concentration, temperature and oxygen content of the reagent, but also on the surface state of the metal. It is the theory of mixed potential developed by Wagner, C, W and Traud W.Z. The electrochemical reactions at the metal–solution interface (electrolyte) are no longer reversible and therefore the Nernst-type equilibrium equation is no longer valid. In these conditions, the open circuit potential is irreversible since the nature of the metal–solution interface varies with time, so we find ourselves in a “non-stationary equilibrium”.

**Figure 5:**
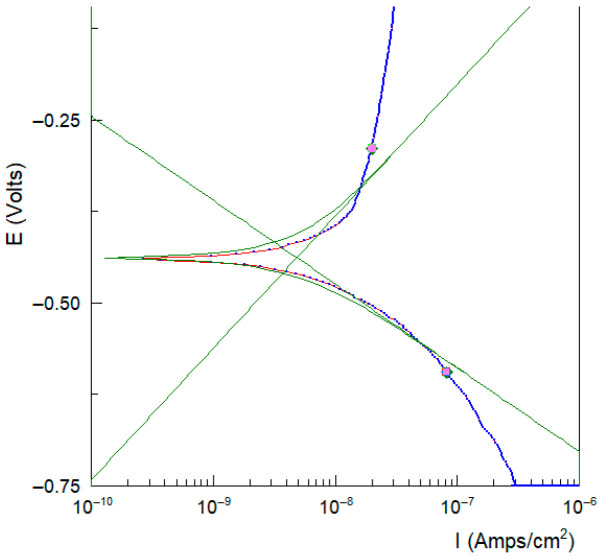
Calculation of i_corr_ and E_corr_ in the Tafel domain for sample #3/SLM, CView routine SCRIBNER.

Thus, to trace the polarization of curves in a non-static regime, it is important to have a long-term immersion. From experience, we plot the polarization curves after 16 h of immersion of the sample in the electrolyte. The scan is in the domain of Tafel Eoc ± 250 mV vs. SCE. Thus we can determine the i_cor_ and E_cor_. We can use two calculation routines, the PAR Calc routine (EG&G PARC Soft Corr) and CView (SCRIBNER Associates Inc., Southern Pines, NC, USA). Figure 5 shows the calculation of icor and ecor by the interactions of anodic and cathodic Tafel slopes for sample #2.

Table 4 summarizes the corrosion potential (E_corr_) and corrosion current density (i_corr_) values for the investigated Co–Cr alloys. All samples exhibit corrosion currents of the same order of magnitude (nA/cm^−2^), indicating a low corrosion rate. However, the additively manufactured samples (SLM and DMLS) show a slightly higher susceptibility to corrosion compared to the casting and CAD/CAM specimens.

**Figure 6:**
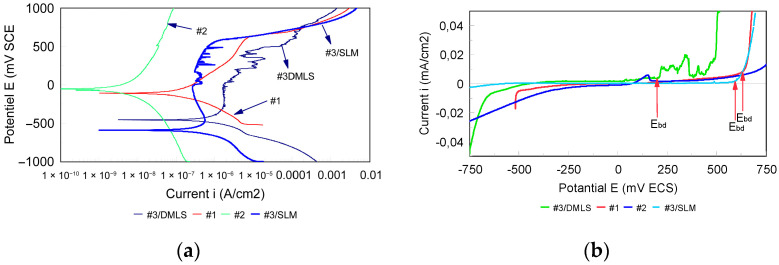
(**a**) Polarization curves in semi-logarithmic axes. (**b**) Polarization curves in linear axes.

The potentiodynamic polarization curves recorded between −1000 mV and +1000 mV vs. SCE are presented in semi-logarithmic scale in Figure 6a. A shift of the curves toward higher current densities is observed for samples #1 (casting), #3 (SLM), and #4 (DMLS) compared to sample #2 (CAD/CAM), indicating increased corrosion activity.

The very low current densities (10^−8^–10^−9^ A/cm^−2^) confirm that generalized corrosion is strongly controlled by passivation. This behavior is characteristic of Co–Cr alloys and is attributed to the formation of a stable chromium-rich oxide film (mainly Cr_2_O_3_), which limits charge transfer at the metal–electrolyte interface. The presence of molybdenum further enhances passive film stability, improving resistance to localized corrosion and delaying passivity breakdown.

Figure 6b presents a linear representation of the polarization curves in the range −750 mV to +750 mV vs. SCE, with current densities between −0.04 and +0.04 mA/cm^−2^. This representation allows identification of the breakdown potential (E_bd_), defined as the potential at which a sharp increase in anodic current occurs.

The potential range between the corrosion potential (E_corr_) and the breakdown potential (E_bd_) defines the passive domain, within which corrosion remains limited. A wider passive domain indicates improved electrochemical stability and reduced susceptibility to accelerated corrosion under polarization or galvanic coupling conditions. The corresponding E_bd_ values are reported in Table 4.

**Figure 7:**
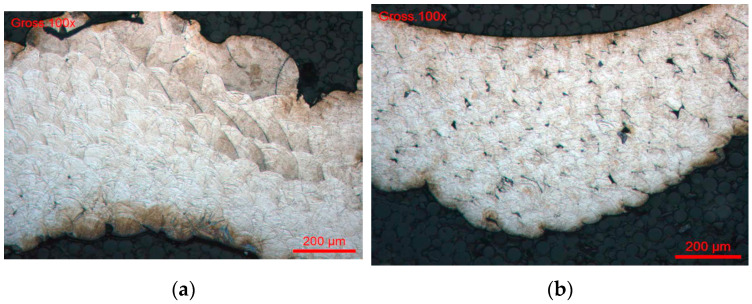
(**a**) Metallographic observation on a vertical cutting. Pile of the layers of CrCo. (**b**) Metallographic observation on a transversal cutting.

The Co–Cr alloy obtained by CAD/CAM machining has the highest electrochemical performance, with a relatively noble corrosion potential and passivity breaking point up to about 740 mV/SCE. This wide passivity domain translates an excellent electrochemical stability, directly related to the observed non-homogenic microstructure. The γ matrix (FCC) is continuous, chemically uniform, and free of marked segregations or secondary phases that may generate galvanic micro-couples. This behavior is attributable to the industrial processes of CAD/CAM disk fabrication (forging, HIP), which promote a dense, thermodynamically stable and weakly stress-free microstructure. The homogeneous distribution of Cr throughout the matrix ensures the formation of a continuous and uniform Cr_2_O_3_ passive film with consistent thickness and composition, which is the fundamental basis for the superior corrosion resistance observed. Unlike cast or additively manufactured alloys, the absence of localized Cr depletion zones means that there are no preferential sites for passive film breakdown, explaining both the wide passivity domain and the low cation release rates of CAD/CAM specimens. In the case of #3/SLM, the structures were examined in vertical and transversal sections (Figure 7a,b).

Figure 7a shows the microstructure of the Co–Cr alloy elaborated by additive manufacturing (SLM/DMLS) observed in the construction direction. A stratified architecture corresponding to the successive layers deposited during the process, well-marked interlayer boundaries and a preferential orientation of the microstructure can clearly be observed, reflecting a marked anisotropy. This lamellar organization results from repeated thermal cycles and high temperature gradients imposed by laser scanning. The interfaces between layers constitute areas with a high density of defects (dislocalities, micro-stresses), which can act as preferential sites for corrosion nucleation, favored paths for ion diffusion, and weakening zones on the passive film.

From an electrochemical point of view, this microstructural anisotropy favors a directional corrosion, dependent on the orientation with respect to the current flow and access of the electrolyte.

Figure 7b shows the microstructure observed perpendicular to the manufacturing direction. It highlights interlayer porosities, “lack-of-fusion” defects and a heterogeneous distribution of molten zones. These defects are characteristic of SLM/DMLS processes when the energy parameters (laser power, scanning speed, overlap) are not perfectly optimized. Porosities promote local retention of the electrolyte, which induces local acidification and leads to premature degradation of the passive film. From an electrochemical point of view, these zones act as micro-anodes, accelerating the local dissolution of cobalt and explaining the higher corrosion current densities and lower passivity breakdown potentials that probably explain the perturbations recorded on the potentiodynamic curves in Figure 7b.

‑ *Localized corrosion, pitting and crevice corrosion*

Crevice corrosion is a form of localized corrosion that develops in confined areas, where oxygen is quickly consumed and the regeneration of the non-solid film is limited. This is due to the pH, which drops locally, and the concentrations of chloride ions. For Co–Cr alloys, the mechanism is dominated by local destabilization of the Cr O film, preferential dissolution of cobalt, and formation of a differential corrosion cell of aeration. A risk factor is the presence of σ/μ/R phases in their microstructure as well as marked interdendritic segregation or grain boundaries chemically depleted in Cr. It is a localized morphology of corrosion that cannot be ignored in dental prosthetic assemblies in Co-Cr alloys.

**Figure 8:**
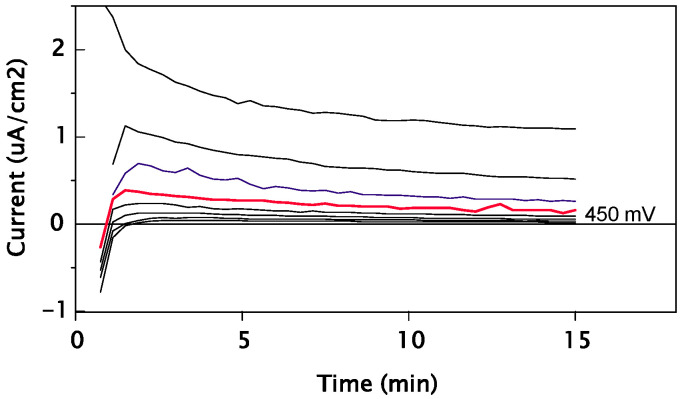
Alloy #2: evolution of current densities at various potentials across the pitting potential Ecrev 450 mV.

Figure 8 displays the evolution (during 15 min) of the current densities under different pre-selected potentials across the critical potential for pitting or crevice corrosion (potentiostatic curves) for sample #2. The potential increment between two curves is 50 mV. The analysis of the results and the optical examination of the surfaces at the base of the collar allowed for a precise determination of the pitting or crevice potentials Ecrev. Table 5 presents the values of the crevice potentials E_crev_ determined for the tested samples.

SLM/DMLS alloys are particularly sensitive to crevice corrosion, as they reveal interlayer interfaces that create internal micro-crevices; porosities trap the electrolyte and form a confined environment that prevents repassivation. It is also necessary to take into consideration their microstructure, which (a) is anisotropic and stratified, (b) has numerous interlayer interfaces, (c) has porosities and melt defects, and (d) has high residual stresses.

The crevice corrosion potential of Co–Cr alloys strongly depends on microstructural homogeneity. CAD/CAM alloys exhibit high crevice corrosion potentials (>450 mV/ECS), whereas cast and additively manufactured alloys show significantly lower values due to segregation and interlayer defects, respectively, leading to early passive film breakdown under confined conditions.

### 3.3. Cation Release

The extraction depends on a great number of parameters—on the one hand, the release environment (chemical composition, pH, chemical stability over time, development of bacteria, temperature, etc.), and on the other hand, the sample parameters and the extraction recipient (geometric shape, surface condition, volume/surface ratio, position of samples during release, etc.). More information for the choice of the environmental conditions is obtained by consulting potential–pH equilibrium diagrams for the chemical element-water system [25]. All these factors generate measurement results with a very large variation from one laboratory to another. Consequently, it is more difficult to obtain close statistical agreement between laboratories when performing such release tests.

The extraction of the Co–Cr samples was carried out in two different media: Medium A (0.07 N HCl, pH = 1.15) and Medium B (0.1% NaF + 0.1% KF, pH = 7.9). Blank solutions corresponding to each medium were used as controls. Four manufacturing processes were investigated: conventional casting, CAD/CAM milling, Selective Laser Melting (SLM), and Direct Metal Laser Sintering (DMLS).

A total of 20 elements were quantified in the extraction solutions using inductively coupled plasma–mass spectrometry (ICP–MS). The concentrations of released elements are expressed in µg/L and are summarized in Table 6.

Table 6 shows the values measured for each sample as well as those of blanks to take into account any release from the extraction recipients. Given that the ratio of volume of the extraction solution/surface of the sample is always equal to one, elements As, Ba, Cd, HF, Hg, Nb, Pb, Sb, Se, Te, V and Zr do not appear in Table 6 because their concentrations have been found to be close to or below the detection limit. They will however be discussed as well. On the other hand, Cu, Ni, and Ti are found in traces in the form of impurities in the CoCr alloys. Copper is a permanent pollution due to the production process that starts from metal waste coming from certain geographical areas. Today, an oxidation process in cast iron tanks is not sufficient, and a second vacuum casting is very expensive.

Table 6 allows a comparative analysis between Medium A (HCl 007N), pH = 1.15, and Medium B (0.1% NaF + 0.1% KF), pH = 7.9, compared to blanks for the casting, CAD/CAM, SLM, and DMLS manufacturing processes. For the base matrix Co, Cr, Mo, and Fe, we proceed with several analyses:

*(a) Environmental effect (calculation of amplification factors A vs. B)* (Table 7):

We calculate an average concentration value for each element in Table 7. Then we proceed with a B/A ratio calculation for each element (Table 7). Table 7 shows the following results:

‑ Chromium (Cr): 25 times more released in an acid medium with a tendency to destroy the passive film Cr O in HCl.
‑ Cobalt (Co): 2.4 times more released in an acid medium with a widespread corrosion trend dominant in the proton environment.
‑ Molybdenum (Mo): 1.4 times higher in a fluorinated medium with an ability to form fluoride complexes.
‑ Tungsten (W): 2.5 times more reactive and mobile in fluorine medium with the possible formation of tungstate/fluoro-tungsten and complexes and increased solubility in fluorine environment; it shows a behavior different from that of Cr and Co (which are dominated by acidity).
‑ Iron (Fe): ~2 times higher in HCl, which provides a global matrix dissolution.
‑ Nikel (Ni): About 1.3 times more relieved in a fluoridated medium but with a moderate effect compared to Cu or W.
‑ Copper (Cu): Three times more released in a fluorinated medium with probable complexation of Cu–F.

*(b) Comparison of processes*

‑ Casting: Moderate-to-fairly high release in an acid medium. Sensitive to selective attacks related to interdendritic segregation.
‑ CAD/CAM: Globally the lowest release, very good stability in a fluorinated medium, homogeneous microstructure and passivation of the slab.
‑ SLM: Intermediate-to-high release and sensitive to interlayer defects. Mo is generally considered as a stabilizing element of the passive film, and is therefore more protective; however, in fluorinated media, Mo can form soluble complexes (molybdates, fluoro-complexes). In other words, molybdenum shows increased mobility in the presence of fluorides, probably due to complexation phenomena, whereas in acidic environments the dissolution remains dominated by cobalt and chromium.
‑ DMLS: Maximum release in HCl, stronger dissolution of Co and Cr and a high microstructural sensitivity.

From a biological perspective, the cation release profiles observed deserve particular attention regarding the two major constituents of the alloy matrix: cobalt and chromium. Cobalt ions (Co^2+^) are known to exert cytotoxic and genotoxic effects at elevated concentrations, including inhibition of cellular respiration, induction of oxidative stress, and potential carcinogenicity as classified by the International Agency for Research on Cancer (IARC Group 2B). Chromium release is of special concern due to the potential for oxidation from the relatively benign trivalent form (Cr^3+^) to the highly toxic hexavalent form (Cr^6+^) under certain biological conditions. The approximately 25-fold amplification of chromium release observed in acidic media is clinically relevant, as patients with gastroesophageal reflux, frequent consumption of acidic beverages, or compromised oral pH may be exposed to significantly elevated Cr concentrations at the prosthesis–tissue interface. While the absolute concentrations measured in the present study remain within the generally accepted biocompatibility thresholds reported in the literature, the selective release patterns observed—particularly the differential behavior of Co and Cr in acidic versus fluoride environments—underscore the importance of considering the patient’s individual oral chemistry when selecting materials and manufacturing routes for long-term prosthetic restorations. It is also important to note that compositional differences between the alloy grades used (particularly the presence of W and variations in Mo content) may contribute to the differential release profiles alongside microstructural effects, and should be considered as a confounding variable in the interpretation of these data.

### 3.4. Statistical Analysis and Non-Parametric Mann–Whitney U Test [69,70]

All quantitative data are expressed as mean ± standard deviation (SD). For each experimental condition (HCl 0.07 N and NaF/KF 0.1% + 0.1%), four independent measurements were considered (n = 4), corresponding to specimens #1, # 2, #3/SLM and #3/DMLS. Blank values corresponding to analytical detection limits were excluded from statistical comparisons. Given the limited sample size and the non-Gaussian distribution observed for several elements (notably Cr and Co in acidic medium), parametric tests were not appropriate; therefore, the non-parametric two-tailed Mann–Whitney U test was selected as the primary inferential method. This test was applied to compare cation release concentrations between the two extraction media (HCl vs. NaF/KF) for each element individually. Statistical significance was set at α = 0.05. All results are reported as mean ± SD throughout. The rationale for choosing a non-parametric approach rests on two considerations: the small sample size (n = 4 per group), which precludes reliable assessment of normality, and the heterogeneous variance observed between groups for several elements (Table 8).

Amplification factors (B/A ratio) were calculated as

$$\mathrm{Amplification}\;\mathrm{factor}=\frac{\mathrm{Mean}\;\mathrm{concentration}\;\mathrm{in}\;\mathrm{NaF}+\mathrm{KF}\;\mathrm{medium}}{\mathrm{Mean}\;\mathrm{concentration}\;\mathrm{in}\;\mathrm{HCl}\;\mathrm{medium}}$$

These ratios were used for mechanistic interpretation but were not subjected to inferential statistical testing. All statistical analyses were performed using standard non-parametric statistical procedures. Due to the small sample size (n = 4 per group), results should be interpreted with caution, and statistical outcomes were primarily used to support mechanistic trends rather than definitive inferential conclusions. Among the analyzed elements, only chromium showed a statistically significant difference between media (*p* < 0.05), confirming the predominant role of proton-induced destabilization of the passive Cr_2_O_3_ film in acidic conditions. Although cobalt showed high mean values in HCl, the difference was not statistically significant due to high variability. Mo and W exhibited amplification factors > 1 in the fluoride medium. Although not statistically significant, the trend indicates preferential fluoride-assisted complexation and partial destabilization of alloy stabilizers.

A comparative analysis of Media A and B based on a non-parametric Mann–Whitney U test was conducted (Table 9).

The results demonstrate the following:

‑ The microstructure plays a determining role in the localization of phenomena.
‑ The acidic environment induces global corrosion controlled by the destabilization of the passive film.
‑ The fluorinated medium does not increase the overall dissolution of the Co–Cr matrix, but promotes selective mobility of secondary elements by complexation.

Table 9 shows that among the analyzed elements, only chromium showed a statistically significant difference between media (*p* < 0.05), confirming the predominant role of proton-induced destabilization of the passive Cr_2_O_3_ film in acidic conditions.

Study Limitations. Several limitations of the present study should be acknowledged. First, three different commercial Co–Cr alloy grades were used across the four manufacturing routes, reflecting clinically relevant material selection but introducing compositional differences as a confounding variable [71]. In particular, variations in tungsten, molybdenum, and chromium content between the alloys may contribute to the differences observed in cation release profiles independently of microstructural effects. Future studies employing a single alloy composition processed by all four manufacturing routes would complement the present findings by isolating the effect of microstructure alone. Second, the sample size (n = 4 per condition) was limited, which constrains the statistical power of inferential analyses; results should therefore be interpreted as supporting mechanistic trends rather than providing definitive conclusions. Third, the extraction conditions (168 h in aggressive media) represent accelerated testing protocols and do not directly replicate the chronic, low-level exposure conditions characteristic of the oral cavity over years of clinical service. Finally, the present study did not include surface roughness measurements or residual stress quantification, both of which may influence passive film stability and corrosion behavior, particularly in additively manufactured specimens. Despite these limitations, the comparative approach employed provides clinically relevant insights into the corrosion behavior of Co–Cr alloys as they are actually used in dental practice.

## 4. Conclusions

This study demonstrates that the corrosion behavior of Co–Cr dental alloys is governed by a complex interaction between microstructure, manufacturing process and oral chemical environment. Integrated analysis of microstructure, generalized corrosion, crevice corrosion sensitivity and multi-elemental cation release establishes a direct link between metallurgical parameters and long-term clinical stability of prosthetic infrastructures produced by conventional casting, CAD/CAM or additive manufacturing (SLM/DMLS).

Although the measured ionic concentrations remain within the accepted thresholds of biocompatibility, the selective release profiles observed in acidic and fluorinated environments highlight the need to preserve the integrity of the passive chromium-rich layer. The preferential release of certain elements, notably cobalt or certain species of chromium, would be clinically relevant in patients predisposed to or repeatedly exposed to fluorinated agents.

The manufacturing pathway appears as a major determinant of electrochemical behavior. Additive manufacturing enables greater customization and geometric complexity, but rapid solidification and microstructural differences can lead to localized corrosion, especially in crevice-like areas.

Future developments of Co–Cr dental biomaterials will likely rely on integrated strategies combining alloy design, microstructural control, and advanced surface engineering. The growing use of digital tools and artificial intelligence may enable predictive modeling of corrosion behavior and support optimized material selection for improved electrochemical stability and biological safety of dental prostheses.

## Figures and Tables

**Table 1:** Chemical composition of the Co–Cr alloys in weight percentage (wt.%) used in evaluation tests.

Code	Process		Co	Cr	W	Mo	Si	Other
#1	Casting	Bulk	62.0	30.0	-	5.4	<1	N, Ta < 1
#2	CAD/CAM	Disk	60.0	28.0	-	9.0	1.0	C, Mn, Fe, Nb < 1
#	/3D Printing /SLM(LPBF) /DMLS	Powder	63.9	24.7	5.0	5.0	1.0	C, Mn, Fe, N < 1

**Table 3:** Phase composition analysis of zones shown in Figure 4b.

Spectrum	C	O	Al	Si	Cr	Mn	Fe	Co	Mo	Total
Spectrum 1—matrix				0.85	28.45		0.85	64.46	5.38	100.00
Spectrum 2—white phase				1.07	38.61		0.38	35.48	24.46	100.00
Spectrum 3—inclusion	14.37	37.13	0.47	17.74	12.26	1.18		14.72	2.13	100.00

**Table 4:** Summary of the electrochemical parameters evaluated from polarization curves.

Alloy	E_corr_	i_corr_	E(I = 0)	E_bd_
	(mV)	nA/cm^2^	(mV)	(mV)
#1	−136	29	−140	625
#2	−43	4.8	−67	740
#3/SLM	−446	420	−580	600
#3/DMLS	−336	410	−450	395

**Table 5:** Crevice corrosion potential Ecrev of tested samples.

Alloy	E_crev_ (mV)
#1	400
#2	450
#3/SLM	350
#3/DMLS	300

**Table 6:** Cations released in extraction solutions above the detection limits of the spectrometers.

Element	A: Blank HCL0.07 N	#1	#2	#3/ SLM	#3/DMLS	B: Blank 0.1% NaF + 0.1% KF	#1	#2	#3/ SLM	#3/ DMLS
	µg/L	µg/L	µg/L	µg/L	µg/L	µg/L	µg/L	µg/L	µg/L	µg/L
Co	0.3	170	180	160	1200	0.4	185	180	150	180
Cr	3.6	36	29	36	1800	4.4	38	29	3.9	6.0
Cu	18	41	42	1.0	2.3	53	43	42	170	2.3
Fe	29.6	50	46.3	191	222	8.0	52	46.3	149	40
Mo	0.2	55	46	13	24	20	50	46	12	89
Ni	2.2	3.5	3.5	1.0	9.7	1.1	3.2	3.5	5.7	11
Ti	0.5	15	11	4.2	7.7	0.2	14	11	19	9
W	0.6	0.6	0.6	2.5	0.5	0.8	0.6	0.6	3.8	5.5

**Table 7:** Calculation of B/A trends.

Element	Average A	Average B	B/A Factor
	µg/L	µg/L	
Co	427.5	173.75	0.41
Cr	475.25	19.7	0.041
Cu	21.6	64.33	2.98
Fe	127.3	71.83	0.56
Ni	4.43	5.85	1.32
Mo	34.5	49.25	1.43
W	1.05	2.63	2.50

**Table 8:** Statistical summary of cation release (µg/L) in HCl (0.07 N) and NaF/KF (0.1% + 0.1%) solutions.

Element	Medium	Mean ± SD (µg/L)	Median	Min–Max	B/A Ratio
Co	HCl	427.5 ± 503	175	160–1200	
	NaF/KF	173.8 ± 16.5	180	150–185	0.41
Cr	HCl	475.3 ± 883	36	29–1800	
	NaF/KF	19.7 ± 16.0	17.5	3.9–38	0.041
Cu	HCl	21.6 ± 23.4	21.7	1.0–42	
	NaF/KF	64.3 ± 68.7	42.5	2.3–170	2.98
Fe	HCl	127.3 ± 88.5	120.5	46.3–222	
	NaF/KF	71.8 ± 49.5	49.2	40–149	0.56
Mo	HCl	34.5 ± 19.5	35	13–55	
	NaF/KF	49.3 ± 32.0	48	12–89	1.43
Ni	HCl	4.43 ± 3.52	3.5	1.0–9.7	
	NaF/KF	5.85 ± 3.28	4.6	3.2–11	1.32
Ti	HCl	9.48 ± 4.62	9.35	4.2–15	
	NaF/KF	13.25 ± 3.91	12.5	9–19	1.40
W	HCl	1.05 ± 0.97	0.6	0.5–2.5	
	NaF/KF	2.63 ± 2.30	2.2	0.5–5.5	2.50

**Table 9:** Mann–Whitney U test results (two-tailed) comparing HCl vs. NaF + KF media.

Element	U Statistic	*p*-Value (Two-Tailed)	Significance (α = 0.05)
Co	6	0.686	not significant
Cr	0	0.029	* not (tendance)
Cu	4	0.343	not significant
Fe	3	0.200	not significant
Mo	5	0.486	not significant
Ni	6	0.686	not significant
Ti	4	0.343	not significant
W	3	0.200	not significant

* The observed differences are trended but not significant.

## Data Availability

The original contributions presented in this study are included in the article. Further inquiries can be directed to the corresponding author.
